# Azvudine (FNC): a promising clinical candidate for COVID-19 treatment

**DOI:** 10.1038/s41392-020-00351-z

**Published:** 2020-10-10

**Authors:** Bin Yu, Junbiao Chang

**Affiliations:** 1 School of Pharmaceutical Sciences, Zhengzhou University, 450001 Zhengzhou, China; 2 Henan Key Laboratory of Organic Functional Molecule and Drug Innovation, College of Life Science and College of Chemistry and Chemical Engineering, Henan Normal University, 453007 Xinxiang, China

**Keywords:** Target identification, Infectious diseases

A very recent work published in *Advanced Science* by our group reveals that 2′-deoxy-2′-*β*-fluoro-4′-azidocytidine (Azvudine, FNC), a clinical candidate originally developed for HIV treatment, has entered clinical trial in China for evaluating its efficacy and safety (ChiCTR2000029853), showing promise for treating novel coronavirus disease 2019 (COVID-19).^1^ This work suggests that nucleoside-based antivirus agents could be repurposed for COVID-19 treatment.

Since the breakout of severe acute respiratory syndrome coronavirus 2 (SARS-COV-2) in December 2019, SARS-COV-2 has spread rapidly. As of August 30, 2020, over 23 million cases have been confirmed worldwide, and more than 0.8 million deaths have been reported. Because of the lack of effective treatment, the fetal cases are increasing globally, particularly in the USA. Therefore, the development of effective vaccines and drugs have been highly pursued for COVID-19 prevention and treatment. To date, some drugs and clinical drug candidates are currently undergoing clinical assessment for treating COVID-19.^2^ Remdesivir, originally developed by Gilead Sciences for treating Ebola virus disease, shows effectiveness in inhibiting the activity of SARS-COV-2 and has received approval in some countries for emergency use in patients with severe COVID-19.^3^ However, rationed supply, side effects, and unsatisfactory phase III clinical results limit the clinical use of remdesivir. In addition, the increasing SARS-COV-2 mutated strains G, GH and GR have posed new challenges for COVID-19 treatment.^4^ Thus, it is urgent to develop safe and effective drugs for treating COVID-19.

**Fig. 1:**
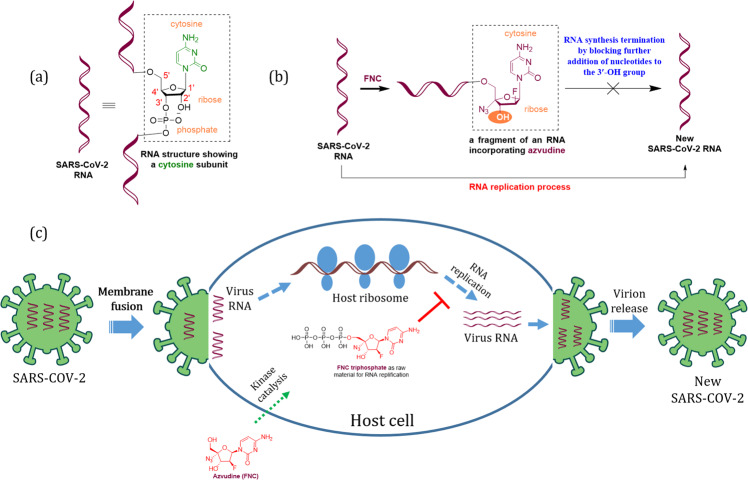
Nucleoside-based anti-HIV clinical candidate azvudine (FNC) for COVID-19 treatment. **a** SARS-COV-2 RNA structure showing a cytosine subunit; **b** A diagram showing the RNA replication process of SARS-COV-2 blocked by FNC; **c** Proposed mechanisms of FNC for inhibiting the activity of SARS-COV-2

SARS-COV-2, a positive-sense single-stranded RNA virus, utilizes nucleosides and nucleotides for RNA synthesis (Fig. 1a). Inside the host cells, nucleoside analogs mimic natural nucleosides and are transformed into corresponding active nucleoside triphosphates through kinase catalysis, which are then embedded in virus RNA during RNA synthesis and then block addition of nucleotides to the 3′-hydroxy group, finally terminating RNA chain synthesis and virus replication (Fig. 1b). Besides, nucleoside-based antiviral agents also inhibit DNA- and RNA-dependent polymerases crucial for viral replication. Therefore, the widely used nucleoside-based antiviral drugs in clinic may have potential for COVID-19 treatment.

FNC, a novel nucleoside-based broad-spectrum anti-virus clinical candidate recently developed in our laboratory for HIV infection treatment and prevention,^5^ is the first dual-targeting nucleoside-based agent that inhibits nucleoside reverse transcriptase and restores expression of cytidine deaminase APOBEC3G (A3G) in HIV-1 patients derived CD4^+^ T cells. The 2′-fluoro group enhances its acidic stability and greatly affects the electronic properties and conformational shape of nucleosides, thus dramatically improving the activities, while modifications at the 4′-position such as introduction of an azido group make the nucleosides adopt an unnatural 3′-C-*endo* conformation, making the compounds being active against HIV and multidrug-resistant HIV strains. FNC has exhibited desirable pharmacokinetics, excellent efficacy and safety in the phase I and II clinical trials (GQ-FNC-2014-2, GQ-FNC-201, and NCT04109183) for treating HIV infection. On March 17, 2020, a phase III clinical trial of FNC was initiated to evaluate the efficacy and safety in HIV-infected treatment naive patients (NCT04303598). On August 12, 2020, the listing application of FNC for HIV treatment was given priority in the review process (CXHS2000016/CXHS2000017). Based on our previous findings, we speculate that FNC could be repurposed for COVID-19 treatment, even for patients infected by mutated SARS-COV-2.

To evaluate the efficacy and safety of FNC in patients with mild and common COVID-19, we recently conducted a randomized, open-label, controlled clinical trial of FNC tablets in China (ChiCTR2000029853).^1^ In this pilot study, 20 patients were randomly assigned to receive FNC or standard antiviral treatment. We found that FNC could shorten the nucleic acid negative conversion (NANC) time. In the FNC group, the mean times of the first NANC is 2.60 d, while for the control group, this value is 5.60 d. The NANC rate is 100% after treatment for 4 d in the FNC group, significantly higher than that (73% after 28 d treatment) in the hydroxychloroquine treatment group. FNC is also efficacious in patients who have received other treatment regimens and could help patients recover faster. Besides, FNC is well tolerated without drug-related adverse events in the FNC group. We speculate that FNC triphosphate could be embedded during RNA synthesis of SARS-COV-2 and inhibits related polymerases, finally leading to RNA replication termination (Fig. 1c). Overall, FNC is a safe and efficacious nucleoside-based drug candidate for treating mild and common COVID-19 and also shows promise for curing severe COVID-19, clinical trials with larger sample size are warranted to further investigate the efficacy and safety of FNC for COVID-19 treatment. The data also suggest that repurposing existing broad-spectrum antivirus agents is a viable and promising strategy for COVID-19 treatment.
